# Attitudes and Practices of Ibero-American Neurosurgeons Toward Psychiatric Comorbidity: A Multicenter Cross-Sectional Study

**DOI:** 10.7759/cureus.109471

**Published:** 2026-05-23

**Authors:** Héctor Aceituno, José Villamediana-Rodríguez, Rosalia Duarte-Ávila, David Pereira-Carpio, Juan J Valero Quintero, José Vásconez-Fabre, Jennifer Vela-Poveda, Elisbeth Gómez, Francisco Rico-Fernández, Juan Lopéz-Urdaneta, Anthuan Hazkour, Luisana Maldonado-De Santiago, René Mena-González, Andrés Rojas, Eugenio Cerezo-Prado

**Affiliations:** 1 Neurological Surgery, Hospital San Juan de Dios de Curicó, Curico, CHL; 2 Neurological Surgery, Hospital San Juan de Dios de Curicó, Curicó, CHL; 3 Neurosurgery, Hospital Camino Universitario Distrital Adelita de Char, Barranquilla, COL; 4 Neurosurgery, Clínica de Bom Jesús, Ponta Delgada, PRT; 5 Neurosurgery, Instituto Clínico La Florida, Caracas, VEN; 6 Neurosurgery, Clínica Miguel Claro, Santiago, CHL; 7 General Medicine, Cesfam Oriente, San Fernando, CHL; 8 Neurosurgery, Hospital Militar Universitario "Dr. Carlos Arvelo", Caracas, VEN; 9 Neurosurgery, Hospital San Juan de Dios de Curicó, Curicó, CHL; 10 Psychiatry, Hospital San Juan de Dios de Curicó, Curicó, CHL

**Keywords:** anxiety, artificial intelligence, attitude of health personnel, depression, latin america, mental health screening, neurosurgery, perioperative care, psychiatric comorbidity, telemedicine

## Abstract

Introduction

Psychiatric comorbidity, particularly anxiety and depression, is associated with worse surgical outcomes across multiple specialties. In Ibero-America, no systematic evidence is available regarding the attitudes and practices of neurosurgeons toward this comorbidity. The present study characterizes such attitudes and self-reported practices and explores their associations with demographic, training-related, and institutional variables.

Methods

We conducted an observational, cross-sectional, multicenter study based on an ad hoc digital questionnaire deployed on LimeSurvey, with parallel Spanish and Portuguese versions, distributed through national neurosurgical societies and digital professional networks between November 2025 and April 2026. The instrument, with content validated qualitatively by a five-expert panel, comprised 37 items organized into four domains: sociodemographic characteristics, attitudes toward mental health, clinical practices and care integration, and professional training and adoption of technological tools. Statistical analysis was performed in Python and included chi-square goodness-of-fit and independence tests, Fisher's exact test, Spearman's correlation, and 95% confidence intervals computed using the Wilson method.

Results

Sixty-three complete responses were obtained from 12 countries (74.6% male; mean age 46.9 ± 9.8 years; median 13 years of practice in the specialty). Of the respondents, 95.2% acknowledged worse surgical outcomes in patients with untreated psychiatric comorbidity, and 90.5% endorsed a level of care equivalent to that afforded to somatic disease; nevertheless, behavior in an equivalent clinical scenario differed significantly (p = 0.002), and the required preoperative psychiatric stabilization time was the only questionnaire item whose distribution did not differ from uniform (p = 0.149). Specific training during residency was absent in 58.7% of participants, and 96.8% were receptive to further education. Institutional availability of neuropsychology units showed a significant regional gradient (χ²₍₄₎ = 15.79; p = 0.003), with the Southern Cone at 8% versus 50-67% in other regions. Acceptance of artificial intelligence and telemedicine tools was high (74.6% and 71.4%, respectively) and was inversely associated with respondent age (ρ = −0.28; p = 0.040 and ρ = −0.30; p = 0.020).

Conclusions

A gap exists between the recognition of the prognostic impact of mental health and the heterogeneity of clinical practice in Ibero-American neurosurgery. This gap is not explained by isolated individual variables but rather by modifiable structural factors. Incorporating dedicated training into residency programs, adopting brief preoperative screening protocols, integrating neuropsychology teams within neurosurgical services, and leveraging digital tools constitute concrete lines of action. Multicenter longitudinal studies with formal instrument validation will allow these findings to be confirmed and extended.

## Introduction

The COVID-19 pandemic has produced a sustained increase in mental health problems, particularly anxiety and depression [1-3]. In Latin America and the Caribbean, a study of 1,338,320 adults documented anxious-depressive symptomatology in four out of every 10 individuals, with a greater incidence in areas of poorer access to health services [4]. The prolonged effects of infection (long COVID) sustain an elevated psychiatric burden up to two years after the initial illness [4], shaping a scenario in which psychiatric comorbidity in the surgical patient is the rule rather than the exception [4].

Mental illness, understood as a clinically significant disturbance in cognition, emotional regulation, or behaviour, usually associated with distress or impairment in important areas of functioning [5], remains stigmatized in healthcare practice. Negative attitudes among health personnel toward patients with mental disorders have been described, translating into delays and lower-quality care, with prevalence varying according to sociocultural factors and level of professional experience [5,6]. International evidence indicates that surgeons and physicians in general tend to distance themselves from these patients, focusing on the physical dimension and neglecting the psychological one, thereby reinforcing the social isolation they already experience [7]. The specialist’s attitude directly influences clinical decision-making, surgical prioritization, and overall quality of care [8,9]. In the neurosurgical setting, this interface is particularly relevant: negative attitudes may manifest as the erroneous attribution of neurological symptoms to psychosomatic causes or as underestimation of reported pain [10].

Depression is characterized by persistently depressed mood or loss of interest and pleasure (anhedonia) lasting at least two weeks, accompanied by neurovegetative and cognitive symptoms - appetite or weight change, insomnia or hypersomnia, psychomotor agitation or retardation, fatigue, feelings of worthlessness or guilt, diminished concentration, and recurrent suicidal ideation, with clinically significant functional impairment [4]. Anxiety involves excessive, persistent fear and worry disproportionate to the situation, expressed through psychic manifestations (apprehension, irritability, concentration and sleep disturbance) and somatic ones (palpitations, dyspnoea, sweating, muscular tension, gastrointestinal distress) [4]. Both conditions demonstrably affect neurosurgical outcomes. Rothrock and colleagues documented, in cervical myelopathy, a significant increase in length of stay and frequency of non-home discharge among patients with a psychiatric history [11].

In spinal surgery, patients with depression and anxiety report greater pain intensity and duration, worse functional outcomes, and more postoperative complications, whereas optimized preoperative mental health - or antidepressant/anxiolytic treatment - equalizes outcomes to those of patients without affective disorder, lending predictive value to preoperative assessment tools [12,13]. Amplification of pain perception has likewise been reported in elective neurosurgical scenarios such as laminectomy, where mood improvement is associated with gains in quality of life [14,15]. A multidisciplinary approach integrating mental health professionals leads to better pain management and lower perioperative anxiety and depression [16].

Despite this evidence, healthcare delivery continues to be dominated by a traditional separation between disciplines that address physiological symptoms and those that attend to psychological ones, with insufficient psychiatric intervention for neurological patients [17]. Reported barriers include the absence of standardized protocols, limited consultation time, poor coordination between specialties, and, importantly, the personal attitudes of the neurosurgeon toward psychiatric comorbidity [7].

Ibero-America is understood here as the supranational cultural and linguistic community comprising the Spanish- and Portuguese-speaking countries of the Americas together with Spain, Portugal and Andorra; the 22 member states recognized by the Ibero-American Conference are Andorra, Argentina, Bolivia, Brazil, Chile, Colombia, Costa Rica, Cuba, the Dominican Republic, Ecuador, El Salvador, Guatemala, Honduras, Mexico, Nicaragua, Panama, Paraguay, Peru, Portugal, Spain, Uruguay, and Venezuela. In this region, where medical training, resource availability, and sociocultural factors differ from those of other settings, no systematic studies have characterized neurosurgeons’ attitudes toward psychiatric comorbidity or their impact on clinical practice [18]. Regional evidence would inform the design of interventions aimed at reducing clinical bias and improving physician-patient communication in this context [18]. Against this background, the aim of the present study is to analyze the attitudes of Ibero-American neurosurgeons toward patients with anxiety and depression and to examine their association with clinical decision-making in neurosurgical practice.

## Materials and methods

Study design

An observational, cross-sectional, exploratory, multicenter study was conducted to characterize the self-reported attitudes and practices of practicing neurosurgeons in Ibero-America toward patients with anxious-depressive comorbidity. The manuscript was prepared in accordance with the CHERRIES recommendations for online surveys and the general principles of the STROBE statement for observational studies.

Study population and recruitment

The target population comprised board-certified neurosurgeons in active practice in Ibero-American countries (Spain, Portugal, and the Spanish- and Portuguese-speaking countries of Latin America). Convenience sampling was performed by means of invitations sent through national neurosurgical societies and the specialty’s digital professional networks. This strategy was preferred over snowball sampling in order to partially mitigate social-proximity bias, although it retains the limitations inherent to non-probability designs, which are discussed below.

Questionnaire distribution was carried out through three complementary channels: (i) institutional emails sent by the executive boards of national neurosurgical societies; (ii) digital professional platforms, principally LinkedIn and ResearchGate; and (iii) announcements at specialty congresses and scientific meetings held during the study period. Data collection was conducted between November 2025 and April 2026.

Given the exploratory, descriptive nature of the study and its multinational scope, no formal a priori sample-size calculation was performed. Recruitment continued throughout the predefined six-month data-collection window, and the final analytic sample was determined by the number of eligible respondents obtained during that period. The resulting sample size supports descriptive estimates with Wilson 95% confidence intervals (CIs) and the detection of large effect sizes in bivariate comparisons, but is acknowledged as a limitation for the detection of smaller effects, in line with the hypothesis-generating purpose of the study.

Eligibility criteria

Eligible participants were professionals holding a specialist degree in neurosurgery, in active clinical practice, with regular activity in Ibero-American countries, and who voluntarily agreed to participate by means of electronic informed consent. Excluded were professionals from specialties other than neurosurgery, neurosurgeons without current clinical activity, those practicing outside the Ibero-American geographical area, and questionnaires with more than 20% missing responses. Only questionnaires completed through the final page of the instrument (page 5 in the LimeSurvey platform) were included in the analysis.

Instrument, content validation, and scoring

An ad hoc digital questionnaire was used, designed specifically for the objectives of the present study by the research team. The preliminary version underwent a content-validation process conducted by an expert panel of five specialists: three board-certified neurosurgeons with academic affiliation, one clinical neuropsychologist, and one researcher with formal training in survey methodology and biostatistics. Experts were selected on the basis of a minimum of five years of clinical or methodological experience in their respective fields. Content validation was conducted qualitatively rather than through quantitative computation of a Content Validity Index: each item of the preliminary version was reviewed independently by every member of the panel; written comments on relevance, clarity, and wording were consolidated, and revisions were incorporated through iterative consensus discussion until the panel agreed that the 37 retained items adequately represented the four target domains. Quantitative metrics of content validity (item-level and scale-level CVI) and other psychometric properties (factor structure, internal consistency, test-retest reliability) were not assessed in the present study and are planned as a separate methodological validation study; this is acknowledged as a limitation. 

The final instrument was deployed on the LimeSurvey platform in parallel Spanish and Portuguese versions. The Portuguese version was produced by forward translation by a Portuguese-speaking neurosurgeon and cross-reviewed by the research team, with category-by-category verification of semantic equivalence between the two language versions. A formal back-translation procedure was not performed and is acknowledged as a limitation of the cross-language equivalence of the instrument. Access to the questionnaire was preceded by a mandatory electronic informed-consent screen. The instrument consisted of 37 structured items organized into four thematic domains: (I) demographic and professional characteristics of the respondent, including training, experience, subspecialty, and practice setting; (II) attitudes and perceptions toward patients with psychiatric comorbidity and their influence on clinical decision-making; (III) diagnostic and screening tools used to detect anxiety and depression in the perioperative period; and (IV) institutional resources and perceived barriers to the comprehensive management of these patients. An additional optional open-text question (free comments) was included at the end of the instrument and was not part of the 37 structured items. Participant anonymity was ensured by configuring the survey to omit personal identifiers.

Scoring Criteria

The 37 structured items were distributed across four response formats: (a) demographic and professional characteristics (items 1-10, n = 10), combining single- and multi-select categorical items, free-text items, and numerical items (age, years of practice, weekly hours, monthly workload as percentages summing to 100), analyzed as descriptive variables and not scored on an ordinal scale; (b) five-point Likert items (items 11-25, 28, 33-37; n = 21), all scored on a 1-5 scale with anchors specific to each item (agreement, frequency, intensity, willingness, perceived outcomes), where higher scores indicate a more favorable attitude, a higher frequency of the behavior assessed, or a greater perceived intensity, depending on the construct; (c) multi-select categorical items (items 26, 27, 30; n = 3), analyzed as the proportion of respondents endorsing each option; and (d) other categorical items (items 29, 31, 32; n = 3), with nominal categorical responses (Yes/No/Maybe; ordered availability categories; Yes/No). All Likert-type items used a five-point response scale ranging from 1 (minimum) to 5 (maximum); for six Likert items (13, 14, 17, 18, 36, 37), an additional non-numerical “don’t know / no response” category was offered and was excluded from ordinal analyses. The complete item-by-item scoring key is provided in Supplementary Files S1 (Spanish), S2 (Portuguese), and S3 (English translation for reader reference).

Subscale Structure

The four thematic domains were used to organize item presentation and the reporting of results, but were not treated as formal psychometric subscales in the present analysis. Items were analyzed individually rather than aggregated into domain scores, because the instrument has not yet undergone factor-analytic confirmation of its internal structure. Consequently, no interpretation ranges (e.g., low/intermediate/high) were derived for any of the four domains; the definition of subscale scores and their interpretation ranges will be addressed in the planned psychometric validation study.

Ethical considerations

The study was conducted in accordance with the principles of the Declaration of Helsinki in its current version. Participation was voluntary, anonymous, and preceded by electronic informed consent, which detailed the study objectives, the unpaid nature of participation, the academic use of the data, and the right to withdraw from the questionnaire at any time without justification. No identifying information was collected from participants, nor were any patient data gathered. The protocol was reviewed and approved by the Scientific Ethics Committee of the Servicio de Salud del Maule (Chile), reference no. 0043-2025.

Statistical analysis

Data were exported from LimeSurvey in spreadsheet format and processed using Python 3.11, with the pandas library (v2.x) for tabular handling, SciPy (v1.x) for hypothesis testing, and statsmodels (v0.14) for the calculation of Wilson CIs. Responses from the Portuguese version were harmonized to the categories of the Spanish version through the same category-by-category semantic-equivalence procedure described in the Instrument subsection, while the original instrument language was retained as a categorical variable for any sensitivity analysis. Country names were normalized by removing orthographic variants, and potential identifiers were removed from the final analytic dataset.

Categorical variables were described using absolute and relative frequencies, accompanied by 95% CIs computed by the Wilson method, which is preferred over the Wald method because of its better coverage when proportions approach the extremes of the interval. Continuous variables were initially evaluated with the Shapiro-Wilk test to assess the normality assumption. Variables with approximately normal distribution were summarized as mean and standard deviation; those with skewed distributions, as median and range.

Univariate analysis of categorical questions was performed using the chi-square goodness-of-fit test, comparing the observed distribution against the null hypothesis of equal distribution among categories. For bivariate analyses, the chi-square test of independence was used, with Yates' continuity correction in 2 × 2 tables when appropriate, and Fisher's exact test in tables with cells of expected frequency below five. Correlations between continuous variables were computed with Spearman's ρ coefficient, given the non-normality of the distributions and its robustness to outliers. Effect sizes were reported as Cramér's V for chi-square associations and as odds ratios with 95% CIs for 2 × 2 contingency tables; for Spearman correlations, the ρ coefficient itself served as the effect-size estimator.

Four bivariate contrasts of interest were planned a priori: institutional availability of a neuropsychology unit by country of practice; training received during residency by respondent gender; the association between years of neurosurgical experience and time devoted to emotional exploration of the patient; and the relationship between self-reported behavior toward controlled psychiatric comorbidity and the perception of worse surgical outcomes in untreated patients. For all inferential procedures conducted in this study - chi-square goodness-of-fit tests, chi-square tests of independence with Yates’ continuity correction, Fisher’s exact test, and Spearman’s ρ correlation - statistical significance was set a priori at p < 0.05, two-tailed. p-values are reported to four decimal places, except when p < 0.001, in which case this convention is stated.

Given the exploratory nature of the study and the limited number of planned comparisons, no correction for multiple comparisons was applied; associations are therefore interpreted as hypothesis-generating. Missing data were not imputed: each question was analyzed over the total of valid responses for that variable, with the corresponding denominator indicated when it differed from the overall sample size.

## Results

Sample characteristics

A total of 63 complete responses were obtained from 12 Ibero-American countries; 58 were completed in the Spanish version and 5 in the Portuguese version, administered to Brazilian neurosurgeons. The geographical distribution, with predominant representation of Chile, Argentina, Spain, and Brazil, together with the age, gender, professional experience, and practice-setting profile of the sample, is summarized in Table 1.

**Table 1:** Sociodemographic and professional characteristics of the sample SD: standard deviation. Percentages were computed over the total of complete responses (N = 63). For multiple-selection variables (subspecialty and practice institution), the sum of percentages may exceed 100%.

Characteristic	N = 63
Survey language, n (%)	
Spanish	58 (92.1)
Portuguese	5 (7.9)
Age, years	
Mean ± SD	46.9 ± 9.8
Median (range)	46 (28–69)
Gender, n (%)	
Male	47 (74.6)
Female	16 (25.4)
Years in medical practice	
Mean ± SD	21.9 ± 10.1
Median (range)	20 (4–45)
Years in neurosurgical practice	
Mean ± SD	15.8 ± 10.3
Median (range)	13 (1–40)
Country of practice, n (%)	
Chile	15 (23.8)
Argentina	10 (15.9)
Spain	8 (12.7)
Brazil	8 (12.7)
Mexico	6 (9.5)
Ecuador	5 (7.9)
Colombia	4 (6.3)
Cuba	3 (4.8)
Bolivia	1 (1.6)
Nicaragua	1 (1.6)
Peru	1 (1.6)
Venezuela	1 (1.6)
Subspecialty (multiple selection), n (%)	
Spine	36 (57.1)
Neuro-oncology	25 (39.7)
Trauma	23 (36.5)
Cerebrovascular	16 (25.4)
Pain	14 (22.2)
Pediatric	10 (15.9)
Other	5 (7.9)
Functional	3 (4.8)
Peripheral nerve	3 (4.8)
Main practice institution (multiple selection), n (%)	
University-affiliated public hospital	33 (52.4)
Independent private clinic	26 (41.3)
Non-university public hospital	16 (25.4)
Non-university private hospital	12 (19.0)
University-affiliated private hospital	8 (12.7)
Military institution	2 (3.2)
Research center	1 (1.6)
Hours per week devoted to neurosurgery	
Mean ± SD	47.3 ± 15.5
Median	45
Surgical procedures per year, n (%)	
Fewer than 30	3 (4.8)
30–60	16 (25.4)
61–120	19 (30.2)
121–250	20 (31.7)
More than 250	5 (7.9)

Overall, the sample comprised mid-career professionals, with a median experience of 13 years in the specialty, a male predominance and a high weekly workload (median 45 hours), who frequently combined work in university-affiliated public hospitals with practice in independent private clinics, in keeping with the dual-employment pattern characteristic of the Ibero-American healthcare system. Most performed an intermediate surgical volume of 61 to 250 procedures per year, with spine, neuro-oncology, and trauma constituting the predominant areas of activity.

Attitudes toward mental health in the neurosurgical setting

The responses corresponding to this domain are shown in Table 2.

**Table 2:** Attitudes toward mental health in the neurosurgical setting (Q11–Q15, Q19) Chi-square goodness-of-fit test against a uniform distribution (H₀: categories are equally distributed). 95% CI computed using the Wilson method. Significance: * p < 0.05; ** p < 0.01; *** p < 0.001. Categories are listed in descending order of frequency.

Question	Response category	n (%)	95% CI	χ² (p)
Q11. Importance of knowing the patient's mental health	Extremely important	43 (68.3)	56.0–78.4	42.3 (< 0.001)***
	Moderately important	19 (30.2)	20.2–42.4	
	Slightly important	1 (1.6)	0.3–8.5	
Q12. Frequency of treating patients with anxiety/depression	Often	28 (44.4)	32.8–56.7	20.0 (< 0.001)***
	Very often	17 (27.0)	17.6–39.0	
	Sometimes	15 (23.8)	15.0–35.6	
	Rarely	3 (4.8)	1.6–13.1	
Q13. Mental illnesses are as important as physical ones	Strongly agree	51 (81.0)	69.6–88.8	106.7 (< 0.001)***
	Agree	8 (12.7)	6.6–23.1	
	Neither agree nor disagree	2 (3.2)	0.9–10.9	
	Strongly disagree	2 (3.2)	0.9–10.9	
Q14. They deserve the same level of care	Strongly agree	45 (71.4)	59.3–81.1	110.3 (< 0.001)***
	Agree	12 (19.0)	11.2–30.4	
	Neither agree nor disagree	3 (4.8)	1.6–13.1	
	Disagree	2 (3.2)	0.9–10.9	
	Strongly disagree	1 (1.6)	0.3–8.5	
Q15. Comfort in treating anxiety/depression	Moderately comfortable	27 (42.9)	31.4–55.1	23.8 (< 0.001)***
	Quite comfortable	21 (33.3)	22.9–45.6	
	Slightly uncomfortable	14 (22.2)	13.7–33.9	
	Uncomfortable	1 (1.6)	0.3–8.5	
Q19. Mood contributes to recovery	Considerably	34 (54.0)	41.8–65.7	27.0 (< 0.001)***
	Extremely	27 (42.9)	31.4–55.1	
	Moderately	2 (3.2)	0.9–10.9	

Recognition of mental health as a clinically relevant dimension was virtually unanimous: when responses of maximal and moderate importance were combined, agreement reached 98.5% (χ²₍₂₎ = 42.29; p < 0.001). Perceived equivalence between mental and physical illness was equally robust, with the proportion of participants strongly agreeing exceeding 80%, and was replicated in the question on equivalent level of care, where combined agreement reached 90.5% (95% CI 80.7-95.6).

This declarative consensus, however, coexisted with less uniform clinical comfort. When asked about their subjective experience treating patients with anxiety or depression compared with those presenting with purely physical pathology, fewer than one-third reported feeling quite comfortable, and almost a quarter acknowledged some degree of discomfort. The frequency with which this type of patient appears in clinical practice was high-more than 70% of respondents see them often or very often-indicating that this is not an occasional scenario. Finally, the appraisal of mood as a prognostic factor for recovery was almost universal: agreement that good mood contributes considerably or extremely exceeded 96%.

Clinical practice, care integration, and institutional resources

The responses for this domain, which articulate self-reported clinical conduct with available institutional resources, are detailed in Table 3.

**Table 3:** Clinical practice, integration, and institutional resources (Q16–Q18, Q20–Q22, Q31–Q34). Chi-square goodness-of-fit test against a uniform distribution. 95% CI computed using the Wilson method. Significance: * p < 0.05; ** p < 0.01; *** p < 0.001. Categories are listed in descending order of frequency.

Question	Response category	n (%)	95% CI	χ² (p)
Q16. Does comorbidity influence the decision to operate?	I request additional evaluation	25 (39.7)	28.5–52.0	14.7 (0.002)**
	I proceed equally in both cases	21 (33.3)	22.9–45.6	
	I postpone until psychiatric optimization	11 (17.5)	10.0–28.6	
	I consider non-surgical options more often	6 (9.5)	4.4–19.3	
Q17. Worse surgical outcomes with untreated comorbidity	Sometimes worse	32 (50.8)	38.8–62.7	23.5 (< 0.001)***
	Yes, usually worse	28 (44.4)	32.8–56.7	
	No difference in outcomes	3 (4.8)	1.6–13.1	
Q18. Required psychiatric stabilization time	4–6 weeks with partial improvement	17 (27.0)	17.6–39.0	8.1 (0.149) ns
	3 months with full remission	14 (22.2)	13.7–33.9	
	Don't know/no response	9 (14.3)	7.7–25.0	
	1–2 weeks with treatment initiation	9 (14.3)	7.7–25.0	
	Operate without delay if indicated	8 (12.7)	6.6–23.1	
	≥ 6 months of demonstrated stability	6 (9.5)	4.4–19.3	
Q20. Self-perceived ability to detect anxiety/depression	Quite capable	36 (57.1)	44.9–68.6	37.4 (< 0.001)***
	Moderately capable	14 (22.2)	13.7–33.9	
	Slightly capable	8 (12.7)	6.6–23.1	
	Extremely capable	5 (7.9)	3.4–17.3	
Q21. Frequency of requesting psychological/psychiatric evaluation	Frequently	27 (42.9)	31.4–55.1	36.3 (< 0.001)***
	Occasionally	19 (30.2)	20.2–42.4	
	Always	12 (19.0)	11.2–30.4	
	Rarely	4 (6.3)	2.5–15.2	
	Never	1 (1.6)	0.3–8.5	
Q22. Communication between Neurosurgery and Mental Health	Often	23 (36.5)	25.7–48.9	23.9 (< 0.001)***
	Rarely	20 (31.7)	21.6–44.0	
	Quite often	11 (17.5)	10.0–28.6	
	Not at all	6 (9.5)	4.4–19.3	
	Always	3 (4.8)	1.6–13.1	
Q31. Availability of the Mental Health team	On patient demand	22 (34.9)	24.3–47.2	28.0 (< 0.001)***
	Occasionally (sometimes)	19 (30.2)	20.2–42.4	
	Monday–Friday, business hours	17 (27.0)	17.6–39.0	
	Never	3 (4.8)	1.6–13.1	
	Available 24/7	2 (3.2)	0.9–10.9	
Q32. Neuropsychology unit at the institution	No	41 (65.1)	52.8–75.7	5.7 (0.017)*
	Yes	22 (34.9)	24.3–47.2	
Q33. Conduct toward a patient without support network	Seeks institutional alternatives (social work, etc.)	29 (46.0)	34.3–58.2	30.6 (< 0.001)***
	Postpones until minimum support is ensured	12 (19.0)	11.2–30.4	
	Informs of risks but operates	11 (17.5)	10.0–28.6	
	Considers non-surgical options preferentially	8 (12.7)	6.6–23.1	
	Does not affect; operates equally	3 (4.8)	1.6–13.1	
Q34. Practice of building patient trust	Considerably	46 (73.0)	61.0–82.4	48.7 (< 0.001)***
	Extremely	15 (23.8)	15.0–35.6	
	Moderately	2 (3.2)	0.9–10.9	

In response to the comparative scenario, two patients with equivalent surgical indication and similar risk, the only difference being a controlled anxious-depressive comorbidity in one of them, self-reported behaviors were significantly dispersed (χ²₍₃₎ = 14.70; p = 0.002). Almost four out of every 10 participants opted to request additional evaluation, one in three reported proceeding identically in both cases, and the rest were divided between postponing surgery until psychiatric stabilization and reconsidering the indication. This heterogeneity is particularly relevant when contrasted with the response on surgical outcomes: 95.2% of respondents acknowledged that patients with untreated psychiatric comorbidity tend to have worse outcomes. There is, therefore, widespread recognition of the prognostic impact of mental health that does not translate into homogeneous clinical conduct.

The question on the minimum psychiatric stabilization time required prior to elective surgery (Q18) was the only item in the questionnaire whose distribution did not differ statistically from uniform (χ²₍₅₎ = 8.14; p = 0.149). The dispersion of responses, ranging from operating without delay when indicated to requiring more than six months of demonstrated stability, reflects a genuine lack of regional clinical consensus and suggests that, in the absence of specific guidelines, time frames are decided on a case-by-case basis under disparate criteria.

Self-perceived competence to detect anxious-depressive symptomatology was moderately favorable, with two-thirds of participants reporting themselves as quite or extremely capable (χ²₍₃₎ = 37.38; p < 0.001). Such declarative confidence, however, coexists with heterogeneous referral practices: systematic referral for psychological or psychiatric evaluation is reported as 'always' by only 19.0% of participants, while communication with the mental health team showed a clearly bimodal distribution, with peaks at 'often' (36.5%) and 'rarely' (31.7%) and only marginal representation of the intermediate category ('quite often', 17.5%). This pattern suggests two distinct institutional realities within the sample rather than a normal distribution of inter-team communication frequency, and is consistent with the regional structural gradient documented in the bivariate analysis below.

Structural difficulties help contextualize these findings. The availability of the mental health team was described by 95.2% of respondents as limited to business hours, contingent on patient demand, or occasional, with only two participants indicating 24/7 availability. The absence of a formal neuropsychology unit was the predominant scenario (65.1% of institutions; χ²₍₁₎ = 5.73; p = 0.017). When facing the specific scenario of a patient without a family or social support network, the predominant strategy was to mobilize institutional alternatives via social services, while the remaining behaviors were distributed among postponing, informing of risks, operating, or reconsidering the indication; only a marginal minority reported that the absence of social support did not affect their decision. The practice of building trust with patients was rated as central by virtually all respondents.

Diagnostic-tool usage and perceived institutional limitations

Despite the favorable attitudes documented in the preceding subsections, the operational use of psychometric screening for anxiety and depression was strikingly limited. More than half of the respondents reported applying no anxiety- or depression-screening tests during the neurosurgical consultation (57.1%; 95% CI 44.9-68.6), prior to surgery (58.7%; 46.4-70.0), or during postoperative follow-up (58.7%; 46.4-70.0). Each of the three distributions departed significantly from uniform (χ²₍₃₎ = 41.95, 40.94, and 67.71, respectively; all p < 0.001), concentrating responses in the ‘not at all’ and ‘a little’ categories. Only between 15.9% and 19.0% of respondents reported applying screening tests at a level of ‘moderately’ or above at any of the three clinical moments evaluated.

Consistent with this picture, 63.5% of respondents reported that no formal psychometric reassessment is carried out at any point of the postoperative follow-up (Q26); among those who do reassess, the most frequent time-points were three months (20.6%) and one month (19.0%). Re-assessment at six months or one year was uncommon (≤ 6.3%).

When asked which specific instruments are used at their institution (Q27), only 7.9% of respondents reported using the PHQ-9, 15.9% the Beck Depression Inventory, 6.3% the MADRS, and 4.8% the Goldberg Anxiety and Depression Scale. Cognitive screens, the Mini-Mental State Examination (28.6%) and the MoCA (9.5%), were more frequently cited than any specific depression or anxiety scale; 23.8% of respondents reported being unaware of which questionnaires are used at their institution, and 39.7% indicated that no instrument is applied. This pattern suggests that, where any psychometric screening occurs in the neurosurgical setting, it tends to address cognitive rather than affective dimensions, despite the explicit clinical focus of the consultation on anxiety and depression as comorbidities.

The perceived limitations for the management of mental-health comorbidity (Q30, multi-select) were dominated by structural rather than attitudinal factors. Lack of training in mental health was cited by 69.8% of respondents (95% CI 57.6-79.8) and lack of time by 63.5%, followed by lack of collaboration between Neurosurgery and Mental Health (47.6%), high workload and stress (44.4%), and complex clinical condition of the patients (34.9%). Stigmatization by staff (27.0%), patient educational level (27.0%), and infrastructural problems (23.8%) constituted a second tier of obstacles. Regulatory barriers and limited nursing collaboration were marginal (6.3% each). Respondents reported a median of three simultaneous perceived limitations (IQR 2-5; range 0-10).

Time devoted to emotional exploration of the patient (Q35) was reported as a continuous variable measured in minutes per week. The distribution was strongly right-skewed (Shapiro-Wilk W = 0.37, p < 0.001), with a median of 20 minutes per week (IQR 5-60; range 0-1000) and a mean of 56.6 ± 132.1 minutes. One quarter of respondents reported devoting 5 minutes or less per week to this activity, while a small minority (4.8%) reported values above 180 minutes per week, accounting for the high standard deviation. The marked dispersion is consistent with the absence of standardized protocols for the psychosocial dimension of the neurosurgical consultation and supports the later use of Q35 in the bivariate analysis as a marker of the time available for the doctor-patient relationship (Table 4).

**Table 4:** Diagnostic-tool usage, perceived limitations, and time devoted to emotional exploration (Q23–Q27, Q30, Q35). Chi-square goodness-of-fit test against a uniform distribution for ordinal items (Q23–Q25); multi-select items (Q26, Q27, Q30) are summarized as the proportion of respondents endorsing each option, with N = 63 as denominator. Q35 is a continuous variable; the Shapiro–Wilk test confirms non-normality of its distribution. 95% CI computed using the Wilson method. Significance: * p < 0.05; ** p < 0.01; *** p < 0.001

Question/category	n (%)	95% CI	χ² (p) / statistic
Q23. Diagnostic tests for anxiety/depression applied in the neurosurgical consultation			
Not at all	36 (57.1)	44.9–68.6	χ²(3) = 41.95 p < 0.001 ***
A little	17 (27.0)	17.6–39.0	
Moderately	8 (12.7)	6.6–23.1	
Considerably	2 (3.2)	0.9–10.9	
Q24. Diagnostic tests applied prior to neurosurgical procedures			
Not at all	37 (58.7)	46.4–70.0	χ²(3) = 40.94 p < 0.001 ***
A little	14 (22.2)	13.7–33.9	
Moderately	6 (9.5)	4.4–19.3	
Considerably	6 (9.5)	4.4–19.3	
Q25. Diagnostic tests used as part of postoperative follow-up			
Not at all	37 (58.7)	46.4–70.0	χ²(4) = 67.71 p < 0.001 ***
A little	15 (23.8)	15.0–35.6	
Moderately	6 (9.5)	4.4–19.3	
Considerably	4 (6.3)	2.5–15.2	
Extremely	1 (1.6)	0.3–8.5	
Q26. Postoperative timing of psychometric re-assessment (multi-select, % of N = 63)			
Not performed	40 (63.5)	51.1–74.3	—
At three months	13 (20.6)	12.5–32.2	
At one month	12 (19.0)	11.2–30.4	
At one year	4 (6.3)	2.5–15.2	
At six months	2 (3.2)	0.9–10.9	
Q27. Specific psychometric instruments used at the institution (multi-select, % of N = 63)			
None applied	25 (39.7)	28.5–52.0	—
Mini-Mental State Examination (MMSE)	18 (28.6)	18.9–40.7	
Unaware of which questionnaires are used	15 (23.8)	15.0–35.6	
Beck Depression Inventory	10 (15.9)	8.9–26.8	
Montreal Cognitive Assessment (MoCA)	6 (9.5)	4.4–19.3	
Patient Health Questionnaire-9 (PHQ-9)	5 (7.9)	3.4–17.3	
Montgomery–Åsberg Depression Rating Scale (MADRS)	4 (6.3)	2.5–15.2	
Goldberg Anxiety and Depression Scale	3 (4.8)	1.6–13.1	
Other	2 (3.2)	0.9–10.9	
Q30. Perceived limitations for mental-health management (multi-select, % of N = 63)			
Lack of training and education in mental health	44 (69.8)	57.6–79.8	—
Lack of time to attend to patients	40 (63.5)	51.1–74.3	
Lack of collaboration NSx – Mental Health	30 (47.6)	35.8–59.7	
High workload and stress in the medical team	28 (44.4)	32.8–56.7	
Complex clinical condition of patients	22 (34.9)	24.3–47.2	
Stigmatization of mental health by staff	17 (27.0)	17.6–39.0	
Patient educational level and understanding	17 (27.0)	17.6–39.0	
Infrastructure problems / available resources	15 (23.8)	15.0–35.6	
Limited nursing collaboration	4 (6.3)	2.5–15.2	
Regulatory or institutional barriers	4 (6.3)	2.5–15.2	
Other	3 (4.8)	1.6–13.1	
Q35. Time devoted to emotional exploration (minutes per week, continuous)			
Median (IQR)	20 (5–60)	—	Shapiro–Wilk W = 0.37 p < 0.001
Mean ± SD	56.6 ± 132.1	—	
Range	0–1000	—	

Professional training and adoption of technological tools

The findings corresponding to this domain are presented in Table 5 and probably constitute the most telling results of the survey.

**Table 5:** Professional training and adoption of technological tools (Q28–Q29, Q36–Q37) Chi-square goodness-of-fit test against a uniform distribution. 95% CI computed using the Wilson method. Significance: * p < 0.05; ** p < 0.01; *** p < 0.001.

Question	Response category	n (%)	95% CI	χ² (p)
Q28. Training during residency	None	37 (58.7)	46.4–70.0	48.7 (< 0.001)***
	Slight	19 (30.2)	20.2–42.4	
	Moderate	5 (7.9)	3.4–17.3	
	Considerable	2 (3.2)	0.9–10.9	
Q29. Wishes to receive further training and resources	Yes	48 (76.2)	64.4–85.0	55.0 (< 0.001)***
	Maybe	13 (20.6)	12.5–32.2	
	No	2 (3.2)	0.9–10.9	
Q36. Use of telemedicine for mental-health follow-up	Agree	29 (46.0)	34.3–58.2	50.0 (< 0.001)***
	Strongly agree	16 (25.4)	16.3–37.3	
	Neither agree nor disagree	7 (11.1)	5.5–21.2	
	Strongly disagree	4 (6.3)	2.5–15.2	
	Don't know / no response	4 (6.3)	2.5–15.2	
	Disagree	3 (4.8)	1.6–13.1	
Q37. Use of AI in diagnosing anxiety/depression	Agree	28 (44.4)	32.8–56.7	53.5 (< 0.001)***
	Strongly agree	19 (30.2)	20.2–42.4	
	Neither agree nor disagree	7 (11.1)	5.5–21.2	
	Disagree	4 (6.3)	2.5–15.2	
	Strongly disagree	3 (4.8)	1.6–13.1	
	Don't know/no response	2 (3.2)	0.9–10.9	

Almost six out of every 10 neurosurgeons reported having received no specific training on the management of anxiety and depression as a surgical comorbidity during their formative period, and a further 30% reported having received only very limited training. The proportion of participants who rated their training as moderate or fairly comprehensive did not reach 12% (χ²₍₃₎ = 48.68; p < 0.001). In keeping with this curricular gap, the demand for training was almost unanimous: when affirmative and undecided responses are combined, 96.8% of the sample is receptive to receiving further training and resources.

The attitude toward technological innovation was likewise favorable. Agreement with the use of telemedicine and follow-up platforms for neurosurgical patients with psychiatric comorbidity stood at 71.4%, and receptiveness to the use of artificial intelligence in the diagnosis of anxiety and depression was even slightly higher (74.6%; 95% CI 62.7-83.7). In both cases, the proportion of respondents in disagreement did not exceed 11%.

Bivariate analysis of associations

Pre-specified associations were explored among demographic, clinical-practice, and institutional-resource characteristics. The summary of the main contrasts, with effect-size estimators (Cramér’s V for chi-square associations and odds ratio with 95% confidence interval for 2 × 2 tables), is presented in Table 6.

**Table 6:** Bivariate analysis: associations among demographic, practice, and resource variables, with effect-size estimators. Tests selected according to the nature of the variables: chi-square test of independence (with Yates’ continuity correction in 2 × 2 tables, or Fisher’s exact test where expected frequencies were < 5) for associations between categorical variables; Spearman’s ρ coefficient for associations involving ordinal or non-normal continuous variables. Cramér’s V is reported for chi-square associations as an effect-size estimator; for Spearman correlations, ρ itself is the effect-size estimator. Odds ratio with 95% CI is reported only where the contingency table is 2 × 2; ‘n/a (k > 2)’ indicates tables larger than 2 × 2 in which OR is not defined. NSx: Neurosurgery; psy: psychiatric/psychological.

Comparison	Test statistic	p	Cramér’s V	OR (95% CI)	Finding
Significant associations (p < 0.05)					
Region of practice vs Neuropsychology unit (Q32)	χ²(4) = 15.79	0.003	0.50 (large)	n/a (k > 2)	Significant
Southern Cone vs rest of sample (Q32)	χ²(1) = 11.33	< 0.001	0.42 (large)	0.08 (0.02–0.38)	Significant
Age vs acceptance of telemedicine (Q36)	ρ = −0.32	0.02	— (ρ)	n/a	Significant
Age vs acceptance of AI (Q37)	ρ = −0.28	0.04	— (ρ)	n/a	Significant
Perceived limitations count (Q30) vs clinical comfort (Q15)	ρ = −0.28	0.028	— (ρ)	n/a	Significant
Borderline trends					
Behaviour toward comorbidity (Q16) vs worse outcomes (Q17)	χ²(6) = 12.49	0.052	0.32 (moderate)	n/a (k > 2)	Trend
Relevant non-significant associations					
Years of NSx experience vs psy evaluations (Q21)	ρ = −0.01	0.954	— (ρ)	n/a	Non-sig.
Years of NSx experience vs clinical comfort (Q15)	ρ = 0.14	0.274	— (ρ)	n/a	Non-sig.
Years of NSx experience vs minutes of emotional exploration (Q35)	ρ = 0.12	0.348	— (ρ)	n/a	Non-sig.
Gender vs training during residency (Q28)	χ²(3) = 4.05	0.256	0.25 (small)	n/a (k > 2)	Non-sig.
Gender vs acceptance of AI (Q37)	χ²(5) = 2.27	0.81	0.19 (small)	n/a (k > 2)	Non-sig.
Spine subspecialty vs psy evaluations (Q21)	χ²(4) = 3.40	0.493	0.23 (small)	n/a (k > 2)	Non-sig.
Neuropsychology availability vs psy evaluations (Q21)	χ²(4) = 3.87	0.424	0.25 (small)	n/a (k > 2)	Non-sig.
Univ.-affiliated public hospital vs Neuropsychology unit	χ²(1) = 0.27	0.606	0.07 (negligible)	1.31 (0.47–3.65)	Non-sig.
Training received (Q28) vs psy evaluations (Q21)	ρ = 0.09	0.499	— (ρ)	n/a	Non-sig.
Training received (Q28) vs clinical comfort (Q15)	ρ = 0.19	0.131	— (ρ)	n/a	Non-sig.

The most robust association concerned the institutional availability of a neuropsychology unit by geographical region (χ²₍₄₎ = 15.79; p = 0.003; Cramér’s V = 0.50). Regional stratification revealed a clear gradient: in the Andean region (Colombia, Ecuador, Peru, Bolivia, Venezuela), 67% of institutions had a neuropsychology unit; in Iberia and Brazil, 50%; in Central and Northern America, 40%; whereas in the Southern Cone (Chile and Argentina) the figure dropped to only 8% (2/25), with both positive cases concentrated in Argentina and none in Chile. The difference between the Southern Cone and the rest of the sample was strongly significant (χ²₍₁₎ = 11.33; p < 0.001; Cramér’s V = 0.42; OR 0.08, 95% CI 0.02-0.38), indicating that practising in the Southern Cone reduces the odds of working in an institution with a neuropsychology unit by approximately 92% compared with the rest of the sample - a regional structural limitation rather than an isolated phenomenon.

Acceptance of technological tools showed a coherent age-related gradient. Younger neurosurgeons reported significantly greater receptiveness to both the use of telemedicine for mental-health follow-up (ρ = −0.32; p = 0.020) and the use of artificial intelligence in the diagnosis of anxiety and depression (ρ = −0.28; p = 0.040). The median age among those who strongly agreed with the use of AI was 43 years, compared with 57 years among those who strongly disagreed. This pattern suggests that the adoption of digital health technologies in neurosurgery may particularly benefit from the gradual incorporation of younger professional cohorts, without necessarily implying ideological resistance among more experienced professionals, whose position was concentrated in the neutral or simple-agreement categories.

A third significant association linked the number of perceived limitations for the management of mental health (Q30, sum of affirmative responses; median 3, range 0-10) with self-reported clinical comfort when treating these patients (ρ = −0.28; p = 0.028). This negative correlation suggests that the discomfort reported when caring for patients with anxiety or depression does not stem from an isolated attitudinal bias, but is associated with the cumulative perception of structural barriers - time, training, interdisciplinary collaboration, infrastructure. Clinical discomfort therefore appears to have an identifiable systemic substrate that is, in principle, modifiable through institutional interventions.

The association between self-reported behaviour toward controlled psychiatric comorbidity (Q16) and the perception of worse surgical outcomes in untreated patients (Q17) showed a trend in the expected direction without reaching conventional significance (χ²₍₆₎ = 12.49; p = 0.052; Cramér’s V = 0.32). Those who perceived worse outcomes tended to request additional evaluation or postpone surgery more frequently, in an association whose moderate effect size and borderline p-value plausibly reflect a power limitation linked to sample size.

Conversely, several pre-specified contrasts yielded no significant associations. Years of neurosurgical experience were not correlated with the frequency of requesting psychiatric evaluation (ρ = −0.01; p = 0.954), with clinical comfort (ρ = 0.14; p = 0.274), or with the time devoted to emotional exploration of the patient (ρ = 0.12; p = 0.348). Respondent gender was not associated with training received during residency (p = 0.256) or with acceptance of AI (p = 0.810). Self-reported subspecialty did not differentiate referral behaviour for psychiatric evaluation (p = 0.493). Particularly noteworthy was the absence of association between institutional availability of neuropsychology and the frequency of requesting psychological or psychiatric evaluation (p = 0.424), as well as between training received during residency and subsequent clinical conduct (Q28 vs Q21: p = 0.499; Q28 vs Q15: p = 0.131). These absences reinforce the interpretation that the documented clinical heterogeneity is transversal to the professional population and is not explained by isolated individual or institutional characteristics.

## Discussion

The paradox of recognition without protocol

The most striking finding is the coexistence of two contradictory facts. Although 95.2% of respondents acknowledge that untreated psychiatric comorbidity worsens surgical outcomes and 90.5% maintain that these patients deserve the same level of care as those with purely somatic disease, behaviour in a concrete clinical scenario is significantly dispersed, and the required psychiatric-stabilization time (Q18) was the only item without a distribution differing from uniform (p = 0.149). Operating immediately for 12.7% versus requiring more than six months of stability for 9.5% reflects the absence of specific guidelines and a case-by-case decision under disparate criteria.

The paradox is not exclusive to neurosurgery: Young and colleagues reported that only 37% of U.S. spine surgeons used preoperative psychological screening, and just one in ten followed a formal protocol [19]; the same dissociation has been documented in cardiac surgery for more than two decades [20] and is still flagged in current European guidelines [21]. What is particular to neurosurgery is the robustness of the underlying evidence: Deshpande and colleagues, in the Michigan Spine Surgery Improvement Collaborative, documented additive adverse effects of anxiety and depression on patient-reported outcomes, readmission, reoperation, and surgical-site infection [22]. Oteri’s systematic review in brain-tumor surgery (27 studies, 2,558 patients) reported preoperative anxiety prevalence up to 89% with measurable impact on quality of life and recovery [23]. Against this background, the 33.3% of respondents proceeding identically in the face of a controlled anxious-depressive comorbidity is not a neutral datum: it is a therapeutic gap that clinical guidelines should help close.

Self-perceived ability and the screening gap

Sixty-five percent of participants rated themselves as quite or extremely capable of detecting anxious-depressive symptomatology (Q20), yet referral for psychological or psychiatric evaluation is reported as systematic ('always') by only 19.0% of respondents, frequent by an additional 42.9%, occasional by 30.2%, and rare or never in a further 7.9% (Q21). This mismatch echoes the meta-analysis by Mitchell and colleagues (41 studies, 50,371 patients), in which non-psychiatric physicians showed a sensitivity of 47.3% to identify depression without validated instruments [24]: brief, validated screening tools, not clinical suspicion alone, are the most reliable route to reducing underdiagnosis.

Two findings from Table 4 sharpen this argument. First, where any screening occurs, it targets cognitive rather than affective dimensions: cognitive screens (MMSE 28.6%; MoCA 9.5%) outnumber specific depression or anxiety scales (Beck 15.9%; PHQ-9 7.9%; MADRS 6.3%; Goldberg 4.8%), and 39.7% apply no instrument. Second, postoperative re-assessment is virtually absent: 63.5% report that no formal psychometric re-evaluation is carried out at any point of follow-up. The implementation gap is therefore not only quantitative (insufficient screening) but qualitative (the wrong instruments at the wrong moments).

The training gap as a parsimonious explanation

A total of 58.7% of participants reported no specific training on the perioperative management of anxiety and depression, and fewer than 12% rated their training as moderate or comprehensive: the curriculum does not systematically include the perioperative management of mental health. U.S. neurosurgical residencies have progressively incorporated curricular content on surgeon well-being since the 2017 ACGME requirement [25], but very few formalize the teaching of clinical management of psychiatric comorbidity in the surgical patient. The most encouraging finding of this study is that the gap is accompanied by an explicit demand: 96.8% of respondents are receptive to receiving further training. Brief competency-based programmes, e-learning modules, or elective rotations through liaison-psychiatry teams could fill the gap without redesigning the technical core of the specialty.

Structural barriers, regional gradient, and clinical comfort

Two-thirds of respondents (65.1%) work in institutions without a formal Neuropsychology unit, and 95.2% describe mental-health team availability as limited. The regional gradient is steep (χ²₍₄₎ = 15.79; p = 0.003; Cramér’s V = 0.50): in the Southern Cone (Chile and Argentina) only 8% of institutions have a Neuropsychology unit, against 67% in the Andean region, 50% in Iberia and Brazil, and 40% in Central and Northern America (Southern Cone vs the rest: OR 0.08, 95% CI 0.02-0.38). This is a regional structural limitation, not an isolated phenomenon, and reflects health-system organization in which neuropsychology is not always a formally covered service and consultation-liaison psychiatry has not consolidated within surgical services as in other Ibero-American systems.

The dual-sector pattern (52.4% in university-affiliated public hospitals; 41.3% in independent private clinics, frequently simultaneously) fragments follow-up and hampers transfer of clinical information across institutions [26]; for the patient with psychiatric comorbidity, this means the psychiatric dimension may be lost between the team that operates and the team that follows up. Tully and Baker's review of depression, anxiety, and post-CABG outcomes argues for systematic preoperative screening with joint multidisciplinary assessment as a necessary condition for psychiatric treatment to translate into better surgical outcomes [27], a conclusion likely extrapolable to neurosurgery.

An additional bivariate finding offers an important explanatory hypothesis: the number of perceived structural limitations correlated negatively with clinical comfort (Spearman ρ = −0.28; p = 0.028), with a median of three simultaneous limitations per respondent. The discomfort reported is therefore neither ideological nor a fixed personality trait but an aggregate indicator of barriers, lack of training (69.8%), lack of time (63.5%), poor interdisciplinary collaboration (47.6%), and workload (44.4%). The implication is operational: if discomfort is structural, it can be modified through institutional interventions.

Equally instructive is what the bivariate analysis did not find. Years of experience were not associated with the frequency of psychiatric evaluations, clinical comfort, or time devoted to emotional exploration. Gender did not differentiate any dimension. Subspecialty did not differentiate behaviour toward psychiatric comorbidity. Particularly notable was the absence of association between existing institutional neuropsychology and the frequency of requesting evaluations (p = 0.424) and between previous training and subsequent clinical conduct (p = 0.499). These absences reinforce that the documented heterogeneity is transversal and cannot be explained by isolated individual or institutional characteristics: explanatory responsibility shifts toward modifiable systemic variables - curriculum, screening protocols, and structural integration of mental-health teams. Finally, almost half of respondents (46.0%) turn to institutional social-work services when facing a patient without a family support network, and only a marginal minority operates without modifying their conduct, intuitively integrating social determinants of recovery, consistent with the DenHeart study (13,443 cardiac patients), in which loneliness was independently associated with an almost three-fold increase in one-year mortality in women and more than double in men [28].

Technology, generational gradient, and integration

Acceptance of technological tools is high (71.4% for telemedicine; 74.6% for AI in diagnosis) and shows a defined generational gradient: respondent age correlated inversely with acceptance of AI (ρ = −0.28; p = 0.040) and telemedicine (ρ = −0.32; p = 0.020), with median age 43 years among those strongly agreeing with AI versus 57 years among those strongly disagreeing. Brief instruments such as the PHQ-9 (Patient Health Questionnaire-9) and the PHQ-2 (Patient Health Questionnaire-2), recommended by the American Heart Association in cardiovascular populations [29], can be incorporated into the neurosurgical consultation without significantly extending consultation time, and digital psychiatry, asynchronous follow-up platforms, digital phenotyping, conversational chatbots, makes it possible to displace part of the psychopathological assessment outside the in-person consultation [30]. Given that the median time devoted to emotional exploration of the patient is only 20 minutes per week (IQR 5-60; one in four respondents reports 5 minutes or less), these tools do not constitute a technological luxury but a concrete response to a documented structural limitation.

Strengths

Four strengths should be considered when interpreting these findings. First, to our knowledge, this is the first study to characterize, with a pan-Ibero-American scope (12 countries), the attitudes and self-reported practices of neurosurgeons toward psychiatric comorbidity, providing a regional baseline that did not previously exist. Second, the parallel Spanish/Portuguese deployment, with harmonization of response categories by direct semantic equivalence and preservation of the original instrument language as a categorical variable, allowed inclusion of Lusophone respondents without semantic drift. Third, the use of an ad hoc instrument specifically designed for a question without a validated regional instrument, complemented by qualitative content validation by a five-expert panel (three neurosurgeons, one clinical neuropsychologist, one survey-methodology researcher), provides face and content coverage of the four target domains while transparently acknowledging the boundary with full psychometric validation. Fourth, the full reporting of inferential statistics, Wilson 95% confidence intervals for every proportion, Cramér’s V and odds ratios as effect-size estimators for chi-square associations, and Spearman correlations with their own effect-size interpretation, allows readers to gauge the robustness of every reported finding independently of the p-values.

Limitations

Several limitations should be acknowledged. First, the cross-sectional design precludes any inference of causality or temporal directionality: the negative correlation between perceived structural limitations and clinical comfort, the regional gradient in Neuropsychology availability, and the age gradient in technological acceptance describe contemporaneous configurations and cannot be interpreted as cause-effect relationships. Longitudinal designs are required to clarify whether modifying structural variables translates into changes in attitudes and behaviour over time. Second, the sample size (63 complete responses) limits statistical power; the association between clinical conduct and the perception of worse outcomes showed a moderate effect size (Cramér’s V = 0.32) and a trend without reaching significance (p = 0.052), plausibly a power limitation. No formal a priori sample-size calculation was performed, in line with the exploratory purpose of the study.

Third, the study is exposed to two distinct response biases. The questionnaire was self-administered and anonymous, which exposes the data to social-desirability bias - likely inflating reported attitudes and receptiveness to training - and to non-response bias, since neurosurgeons more interested in mental-health topics may have been more inclined to participate; both biases operate in the same direction (overestimation of favorable attitudes) and should be considered when interpreting the magnitude, although not the structure, of the reported proportions. Fourth, the geographical distribution of the sample is uneven: Chile (23.8%), Argentina (15.9%), Spain (12.7%), and Brazil (12.7%) jointly account for 65.1% of respondents, whereas seven of the twelve participating countries are represented by three respondents or fewer. Country-specific inferences are therefore not feasible, and the regional pattern documented for the Southern Cone, although statistically robust, should be interpreted in light of this distribution. Lusophone representation (n = 5) was considerably smaller than Spanish-speaking representation, so inferences for the Lusophone region should be made with particular caution.

Fifth, this is a self-report study, without verification of the practices described or audit of clinical records, so the data reflect attitudes and self-reported behaviours rather than observed conduct. Non-probability convenience sampling through neurosurgical societies introduces selection bias toward professionals with greater academic interest or institutional involvement, which probably overestimates receptiveness to training and to technological tools. Finally, the questionnaire underwent qualitative content validation, but no formal psychometric validation (item-level or scale-level Content Validity Index quantification, factor analysis, or internal-consistency and test-retest reliability assessment). Formal psychometric validation, including the definition of subscale scores and their interpretation ranges, is planned as a separate methodological study.

Implications and future directions

Three lines of action follow from these findings. At the educational level, neurosurgical residency programmes in the region should incorporate specific modules on the perioperative management of anxiety and depression, in line with the explicit demand of the professionals themselves. At the clinical-institutional level, development and regional validation of a brief preoperative screening protocol - adapting instruments such as the PHQ-2 or the HADS (Hospital Anxiety and Depression Scale) to the neurosurgical workflow - would reduce the heterogeneity of criteria documented, especially regarding psychiatric stabilization time prior to elective surgery. At the care-delivery level, formal integration of neuropsychology and consultation-liaison psychiatry teams within neurosurgical services constitutes a priority [31], particularly in the Southern Cone where this figure is structurally absent (Table 6). Asynchronous digital tools may offer a pragmatic complementary route to address both the consultation-time barrier and the declared receptiveness to technological solutions. Future multicenter studies with formal instrument validation, larger sample sizes, and longitudinal design could confirm and extend these findings and assess the impact of specific educational and protocol-based interventions on the clinical outcomes of neurosurgical patients with psychiatric comorbidity.

## Conclusions

This first Ibero-American multicenter study documents a clinically relevant dissociation between neurosurgeons’ virtually unanimous recognition of the prognostic weight of psychiatric comorbidity - 95.2% acknowledge it worsens surgical outcomes and 90.5% endorse a standard of care equivalent to that for somatic disease - and a markedly heterogeneous clinical conduct, the decision regarding preoperative psychiatric stabilization time being the only item without departure from uniform distribution. Because this attitude-practice gap is not explained by individual professional variables nor by the isolated availability of neuropsychology, its origin appears systemic and therefore amenable to structural rather than individual remediation.

Three convergent axes orient the response. A self-acknowledged training deficit - 58.7% received no residency instruction on anxiety and depression, yet 96.8% are receptive to further training - creates fertile ground for competency-based curricular modules and psychiatry liaison rotations. A marked regional gradient in neuropsychology availability (Southern Cone below 10% versus above 50% elsewhere) demands targeted integration of mental-health teams in systems where this resource is structurally absent. The favorable disposition toward telemedicine (71.4%) and AI-assisted screening (74.6%), with a defined generational gradient, suggests that asynchronous digital platforms may operationally compensate for the documented constraint of consultation time (median 20 min/week for emotional exploration). Regional validation of a brief preoperative screening instrument, guidelines that homogenize criteria for psychiatric stabilization, focused educational programs, and multicenter longitudinal designs with formal instrument validation constitute immediate priorities.

## Appendices

Survey instrument - English translation (for reader reference)

Note. This document is a faithful English translation of the final survey instrument actually administered to participants in Spanish and Portuguese. The Spanish and Portuguese versions are the reference instruments effectively deployed via LimeSurvey to neurosurgeons across Ibero-America; this English version is provided for the convenience of English-speaking readers and faithfully reproduces the items, response categories, and ordering of the administered instrument. Where minor differences exist between Spanish and Portuguese wording, response categories were harmonized through direct semantic equivalence as described in the Methods section of the main manuscript.

Informed Consent for Participation in Research on the Attitude of Neurosurgeons Toward Patients with Mental Health Conditions

Principal investigator: Dr. Héctor Aceituno. Telephone: +56 9 4498 3377. Email: haceituno@hospitalcurico.cl

Introduction: Climate change and the COVID-19 pandemic have increased the number of people experiencing mental health problems. As neurosurgeons, we face the challenge of caring for patients with anxiety and depression, which may potentially influence the outcomes of our surgical procedures.

Objective: To evaluate the attitude of neurosurgeons in Ibero-America toward problems of anxiety and depression in their patients, with particular emphasis on how this attitude directly shapes the quality of medical care delivered. This study seeks to identify factors that optimize care processes and improve clinical outcomes for patients with mental health comorbidities.

Procedures: We have designed a survey to explore how neurosurgeons in Ibero-America approach patients with mental health problems. The survey is divided into four sections: (i) Demographic and professional characteristics of participants; (ii) Attitudes toward mental health conditions in neurosurgical patients and their influence on care and decision-making; (iii) Diagnostic tools used to evaluate the mental health of patients; (iv) Professional and institutional resources and limitations. The survey will take no more than 8 minutes to complete.

Benefits: This study will offer a perspective on the views of neurosurgeons regarding patients with anxiety and depression. Based on the results, recommendations may be formulated to optimize the care of these patients and the efficacy of procedures. The benefits of this study are primarily projective and will not have an immediate impact on your current professional practice.

Possible discomforts: Participation in this survey may generate some unease when reflecting on your own professional practices or when questioning aspects of your training and experience. If you experience any emotional distress while completing the survey, we recommend taking a moment to reflect and decide whether you wish to continue. You may also contact the research team for guidance.

Risks: Participation in this research does not entail significant risks to your physical or mental integrity. Data are stored confidentially on a secure server, with encryption, strong password access control, and two-factor authentication.

Costs and compensation: There will be no financial costs for participants, and participation does not entail financial remuneration.

Confidentiality: This survey guarantees the confidentiality of the data collected. It complies with Chilean Law No. 19.628 on the Protection of Private Life and the European Union's General Data Protection Regulation (GDPR). No IP addresses, names, or email addresses are stored. Personal data of participants is de-identified. All information is anonymous and available only to the researchers of this study. Custody of the data is the responsibility of the principal investigator, and data will be retained for 5 years, after which all collected information will be securely deleted.

Potential use of research results: The results will not be used for commercial purposes. They will be presented at national and international congresses and published in indexed peer-reviewed scientific journals.

Voluntary withdrawal from the study: Your participation in this study is completely voluntary. You may decide to withdraw at any time without the need to provide explanations and without negative consequences. A specific revocation form is available by request at haceituno@hospitalcurico.cl.

Sources of funding. This study is funded with the researchers' own resources.

Do you agree to participate in the survey?

☐  Yes

☐  No

Section I. Demographic and Professional Characteristics

1. Gender:

☐  Female

☐  Male

☐  Non-binary

☐  Prefer not to say

2. Age (years):

Age:    ____________________

3. Country of neurosurgical practice:

Country:    ____________________

4. Years of practice as a physician:

Years:    ____________________

5. Years of practice in the specialty of Neurosurgery:

Years:    ____________________

6. Subspecialty of greatest dedication in Neurosurgery (multiple selection allowed):

☐  Cerebrovascular

☐  Neuro-oncology

☐  Spine

☐  Trauma

☐  Functional and stereotactic

☐  Pain

☐  Pediatric

☐  Peripheral nerve

☐  Other:    ____________

7. Select the institution where you primarily work (multiple selection allowed):

☐  University-affiliated public hospital

☐  Non-university public hospital

☐  University-affiliated private hospital

☐  Non-university private hospital

☐  Independent private clinic

☐  Military institution

☐  Research center

☐  Other:    ____________

8. Hours dedicated to neurosurgery per week:

Hours/week:    ____________________

9. Monthly workload distribution (must sum to 100%):

☐  Outpatient consultation: ______%

☐  Hospitalization: ______%

☐  Surgery: ______%

☐  Administrative activity: ______%

☐  Research: ______%

10. Number of surgical procedures performed per year:

☐  Fewer than 30

☐  30-60

☐  61-120

☐  121-250

☐  More than 250

Section II. Attitudes

11. Do you consider it important to know the mental health condition of your patients?

☐  Not at all important

☐  Slightly important

☐  Moderately important

☐  Quite important

☐  Extremely important

12. How often do you treat patients with pre-existing anxiety or depression conditions?

☐  Never

☐  Rarely

☐  Sometimes

☐  Often

☐  Very often

13. Do you believe that mental health conditions are equally important as physical illnesses?

☐  Strongly disagree

☐  Disagree

☐  Neither agree nor disagree

☐  Agree

☐  Strongly agree

☐  Don't know / no response

14. Do you consider that patients with mental health conditions deserve the same level of care as patients with physical illnesses?

☐  Strongly disagree

☐  Disagree

☐  Neither agree nor disagree

☐  Agree

☐  Strongly agree

☐  Don't know / no response

15. How do you feel when treating patients with anxiety or depression compared with patients with physical illnesses?

☐  Uncomfortable

☐  Slightly comfortable

☐  Moderately comfortable

☐  Quite comfortable

☐  Extremely comfortable

16. Suppose you have two patients with equivalent surgical indications and similar risk, the only difference being that one of them presents a controlled anxiety or depression condition. What is your behaviour toward the patient with the controlled psychiatric comorbidity?

☐  I request additional evaluation of the patient with anxiety/depression

☐  I proceed equally in both cases

☐  I postpone surgery until psychiatric optimization

☐  I consider non-surgical options more frequently

17. In your experience, do patients with psychiatric comorbidity tend to have worse surgical outcomes compared with those without mental illness?

☐  Yes, outcomes are usually worse

☐  Outcomes are sometimes worse

☐  No difference in outcomes

18. In your view, what is the minimum psychiatric stabilization time that an anxiety or depression problem should achieve before proceeding with elective surgical management?

☐  Not necessary; I operate without delay if there is a surgical indication

☐  1-2 weeks with treatment initiation

☐  4-6 weeks with partial clinical improvement

☐  3 months with complete remission of symptoms

☐  ≥ 6 months of demonstrated stability

☐  Don't know / no response

19. To what extent do you consider that the patient's good mood contributes to recovery?

☐  Not at all

☐  Slightly

☐  Moderately

☐  Considerably

☐  Extremely

Section III. Diagnostic Tools

20. Do you feel technically capable of suspecting an anxiety or depression problem in the patients you attend?

☐  Not at all capable

☐  Slightly capable

☐  Moderately capable

☐  Quite capable

☐  Extremely capable

21. Are evaluations by psychologists or psychiatrists requested for patients in whom anxiety or depression is suspected?

☐  Never

☐  Rarely

☐  Occasionally

☐  Frequently

☐  Always

22. How often is communication maintained between the mental health team and the neurosurgical specialists?

☐  Not at all

☐  Rarely

☐  Often

☐  Quite often

☐  Always

23. Are diagnostic tests for anxiety and depression applied in the neurosurgical consultation?

☐  Not at all

☐  A little

☐  Moderately

☐  Considerably

☐  Extremely

24. Are diagnostic tests for anxiety and depression applied prior to the performance of neurosurgical procedures?

☐  Not at all

☐  A little

☐  Moderately

☐  Considerably

☐  Extremely

25. Are diagnostic tests for anxiety and depression used as part of the follow-up of postoperative patients?

☐  Not at all

☐  A little

☐  Moderately

☐  Considerably

☐  Extremely

26. If applicable, how many months after the procedure are these tests performed? (Mark all that apply)

☐  At one month

☐  At 3 months

☐  At 6 months

☐  At one year

☐  Not performed

27. Which of these tests are applied at your institution? (Mark all that apply)

☐  Patient Health Questionnaire-9 (PHQ-9)

☐  Goldberg Anxiety and Depression Scale

☐  Beck Depression Inventory

☐  Mini-Mental State Examination (MMSE)

☐  Montreal Cognitive Assessment (MoCA)

☐  Montgomery-Åsberg Depression Rating Scale (MADRS)

☐  Other:    ____________

☐  Unaware of which questionnaires are used

☐  None is applied

Section IV. Professional and Institutional Resources and Limitations

28. Were you taught to address anxiety and depression problems as comorbidities in your patients during your training as a neurosurgeon?

☐  Not at all

☐  Slightly

☐  Moderately

☐  Considerably

☐  Extensively

29. Would you like to receive further training and resources to treat patients with anxiety and depression in your professional setting?

☐  Yes

☐  No

☐  Maybe

30. Which do you consider to be the limitations for the management of patients with mental illness in the field of neurosurgery? (Mark all that apply)

☐  Lack of training and education in mental health

☐  Lack of time to adequately attend to patients

☐  High workload and stress in the medical team

☐  Lack of collaboration between the Neurosurgery service and the Mental Health team

☐  Stigmatization of mental health problems by staff

☐  Educational level and understanding of patients

☐  Complex clinical condition of the patients

☐  Limited collaboration on the part of nursing staff

☐  Regulatory or institutional barriers

☐  Problems of infrastructure and available resources

☐  Other (please specify):    ____________

31. What is the availability of Mental Health teams for your patients?

☐  Never

☐  Occasionally (sometimes)

☐  On patient demand

☐  Monday to Friday, business hours

☐  Available 24 hours a day, 7 days a week (24/7)

32. Does your institution have a Neuropsychology unit that provides services to Neurosurgery?

☐  Yes

☐  No

33. When facing the case of a neurosurgical patient without a support network (family or social), how does this circumstance influence your decision regarding surgery?

☐  It does not affect; I operate equally

☐  I inform the patient of the risks, but I operate

☐  I seek institutional alternatives (social work, etc.)

☐  I consider non-surgical options preferentially

☐  I postpone surgery until minimum support is ensured

34. In your professional activity, do you practice building trust with your patients?

☐  Not at all

☐  Slightly

☐  Moderately

☐  Considerably

☐  Extremely

35. On average, how many minutes per week do you devote to the emotional exploration of your patients (i.e., to listening to and exploring the affective, mood-related, or psychosocial dimension of their condition)?

Minutes per week:    ____________________

36. Could you use technological tools, telemedicine, and follow-up platforms in neurosurgical patients with mental health conditions?

☐  Strongly disagree

☐  Disagree

☐  Neither agree nor disagree

☐  Agree

☐  Strongly agree

☐  Don't know / no response

37. Could you use Artificial Intelligence (AI) tools in your professional activity for the diagnosis of patients with anxiety and depression?

☐  Strongly disagree

☐  Disagree

☐  Neither agree nor disagree

☐  Agree

☐  Strongly agree

☐  Don't know / no response

38. Comments regarding the topic (optional):

____________________________________________________________

____________________________________________________________

If you wish to receive the publications of this study, please indicate your email: ____________________

Thank you for your participation.
